# Using Mobile Phone Data to Predict the Spatial Spread of Cholera

**DOI:** 10.1038/srep08923

**Published:** 2015-03-09

**Authors:** Linus Bengtsson, Jean Gaudart, Xin Lu, Sandra Moore, Erik Wetter, Kankoe Sallah, Stanislas Rebaudet, Renaud Piarroux

**Affiliations:** 1 Department of Public Health Sciences. Karolinska Institutet, Stockholm, Sweden; 2 Flowminder Foundation, Stockholm, Sweden; 3 Aix-Marseille University, UMR 912 SESSTIM (INSERM-IRD-AMU), Marseille, France; 4 College of Information System and Management, National University of Defence Technology, Changsha, China; 5 Aix-Marseille University, UMR MD 3, Marseille, France; 6 Stockholm School of Economics, Stockholm, Sweden

## Abstract

Effective response to infectious disease epidemics requires focused control measures in areas predicted to be at high risk of new outbreaks. We aimed to test whether mobile operator data could predict the early spatial evolution of the 2010 Haiti cholera epidemic. Daily case data were analysed for 78 study areas from October 16 to December 16, 2010. Movements of 2.9 million anonymous mobile phone SIM cards were used to create a national mobility network. Two gravity models of population mobility were implemented for comparison. Both were optimized based on the complete retrospective epidemic data, available only after the end of the epidemic spread. Risk of an area experiencing an outbreak within seven days showed strong dose-response relationship with the mobile phone-based infectious pressure estimates. The mobile phone-based model performed better (AUC 0.79) than the retrospectively optimized gravity models (AUC 0.66 and 0.74, respectively). Infectious pressure at outbreak onset was significantly correlated with reported cholera cases during the first ten days of the epidemic (p < 0.05). Mobile operator data is a highly promising data source for improving preparedness and response efforts during cholera outbreaks. Findings may be particularly important for containment efforts of emerging infectious diseases, including high-mortality influenza strains.

Re-occurring infectious disease outbreaks due to cholera, measles and other preventable infectious diseases contribute to a major disease burden affecting low- and middle-income countries^1^,^2^. Concurrently, outbreaks of new infectious diseases with pandemic potential pose a considerable threat to human life and development^3^,^4^. Response to, and ideally containment of^5^, an infectious disease outbreak can be greatly improved if health care response and outbreak control measures can be focused to areas predicted to be at the highest risk of experiencing new outbreaks^6^,^7^. Accurate models of the geographic distribution of epidemic risk could significantly enhance the population-level effects of interventions implemented to control the spread of transmittable diseases^8^. Considerable progress has been made in predicting temporal evolution of epidemics once outbreaks have progressed beyond a small initial group of cases^7^,^9^. However, predicting spatial transmission routes of epidemics has proven to be remarkably difficult, due to the importance of rare, long-distance transmission events^10^, limited data on population mobility, unknown population immunity levels^9^, low sensitivity and specificity of case reports^11^ and limited access to accurate and spatiotemporally resolved case data^12^.

Empirical data has provided key insight into the spatial spread of measles in England^13^ and Niger^14^ as well as into influenza spread in the USA and Europe^11^,^12^,^15^. While population mobility plays a key role in such modelling studies^10^,^16^, it has not been possible, until now, to study detailed and concurrent data on both population mobility and spatiotemporal distribution of cases. Instead, empirical studies have used either models of population mobility, preferentially gravity models^17^, or census data on work-home commuting as proxies for total mobility during outbreaks^11^. Although highly significant correlations exist between these mobility patterns and retrospective data on epidemic spread, large unexplained variations remain^10^,^18^,^19^. It is also not clear how to choose and properly parameterize mobility models across contexts in new outbreaks. This is especially problematic during the critical early outbreak phases, when interventions have the greatest effect, but limited data are available to fit transmission models.

Anonymous mobile operator data may provide a new source of large-scale empirical data on which to build more accurate models of infectious disease spread. Mobile phone operators register the mobile phone tower closest to the mobile user at the time of each call and text message. This allows individual phones to be localized at a resolution equal to the coverage area of the mobile phone tower (typically one to ten km^2^). In the public health field, this data has notably been used as a proxy for nationwide mobility patterns in malaria modelling studies^20^,^21^. However, the extent to which this type of data accurately reflect movements of infectious persons and its utility in predicting spatial spread of infectious agents have not been evaluated.

The largest cholera epidemic to strike a single country in recent history was the 2010 Haitian outbreak^22^. The first confirmed cholera case in Haiti developed symptoms on October 14, 2010 in a hamlet 60 km north of the capital Port-au-Prince. The epidemic spread first explosively along the nearby Artibonite river (Fig. 1) and subsequently, during a period of two months, throughout the entire country^23^.

The 2010 Haiti cholera epidemic provides a unique opportunity to explore the influence of population mobility on the spatial evolution of a large-scale cholera outbreak. First, cholera had not previously affected the country for at least a century, thereby rendering epidemic development unbiased by differential population immunity. Second, the circumstances and location of the onset of the cholera epidemic are well understood^23^,^24^,^25^. Third, daily case reporting based on WHO criteria was initiated very early throughout the country, and the notification system was highly effective^22^,^23^,^24^,^25^.

In this study, we utilized data on the movement of 2.9 million anonymised mobile phone SIM cards in Haiti during the early phase of the Haitian cholera epidemic together with highly spatiotemporally resolved case data. We used these data to test the hypothesis that mobile operator data could be used to dynamically predict the spatial evolution of the epidemic from outbreak onset.

## Methods

### Data collection

#### Cholera case data

As soon as the epidemic was recognized, the Haitian government, with support from the US Centers for Disease Control and Prevention, implemented a nationwide monitoring program^22^. Each day, government and non-governmental health facilities in Haiti reported probable cholera cases (ambulatory patients, hospital admissions and deaths) to the Directorate of Health in each of the ten administrative departments (Haitian provinces). Probable cases were defined according to a modified WHO definition as “acute watery diarrhoea, with or without vomiting, in persons of all ages”^22^. *Vibrio cholerae* O1 infection was confirmed via bacterial culture for early cases in all departments. We have previously validated case data from the National Cholera Surveillance system by carrying out field investigations, comparing case reports with data available from registers in cholera treatment centres managed by Haitian teams, Doctors without Borders and medical brigades from Cuba^23^.

The daily case reports per health facility enabled us to determine daily case numbers per commune while the epidemic spread throughout the country (October 14 and 64 days onwards). We defined the end of the study period as December 16, when the peak of the epidemic was reached and all but one commune had reported at least one case. In 62 communes out of 140 communes, including the eight communes within the Port-au-Prince metropolitan area, there may have been patients who sought healthcare in neighbouring communes. For all such suspected communes we merged the communes into a single area, thereby creating a total of 78 study areas throughout the country (see S1 for details). One study area was excluded due to absence of mobile network coverage. To predict spatial spread of cholera, we defined a study area to have acquired a novel local outbreak if five or more cases were recorded on any given day, thereby avoiding misclassification of spurious cases of diarrhea as new cholera outbreaks. For sensitivity analyses of the outbreak definition, see S5.

#### Mobile phone data

The analysed anonymous mobile phone data consisted of the last outgoing call or text message each day from October 15 to December 19, 2010 for all 2.9 million users belonging to the largest mobile operator, Digicel Haiti^26^. Research on bias in population mobility estimates stemming from differential ownership of mobile phones between socio-economic groups has been evaluated in Kenya, finding only minor bias^27^. Mobile phone mobility patterns based on the mobile phone dataset used in this study have previously been shown to approximate mobility patterns from a representative survey of 2,500 households in the capital Port-au-Prince, Haiti, during the same year (2010)^28^. S2 provides further details on the mobile operator dataset.

### Analyses

**Figure 1:**
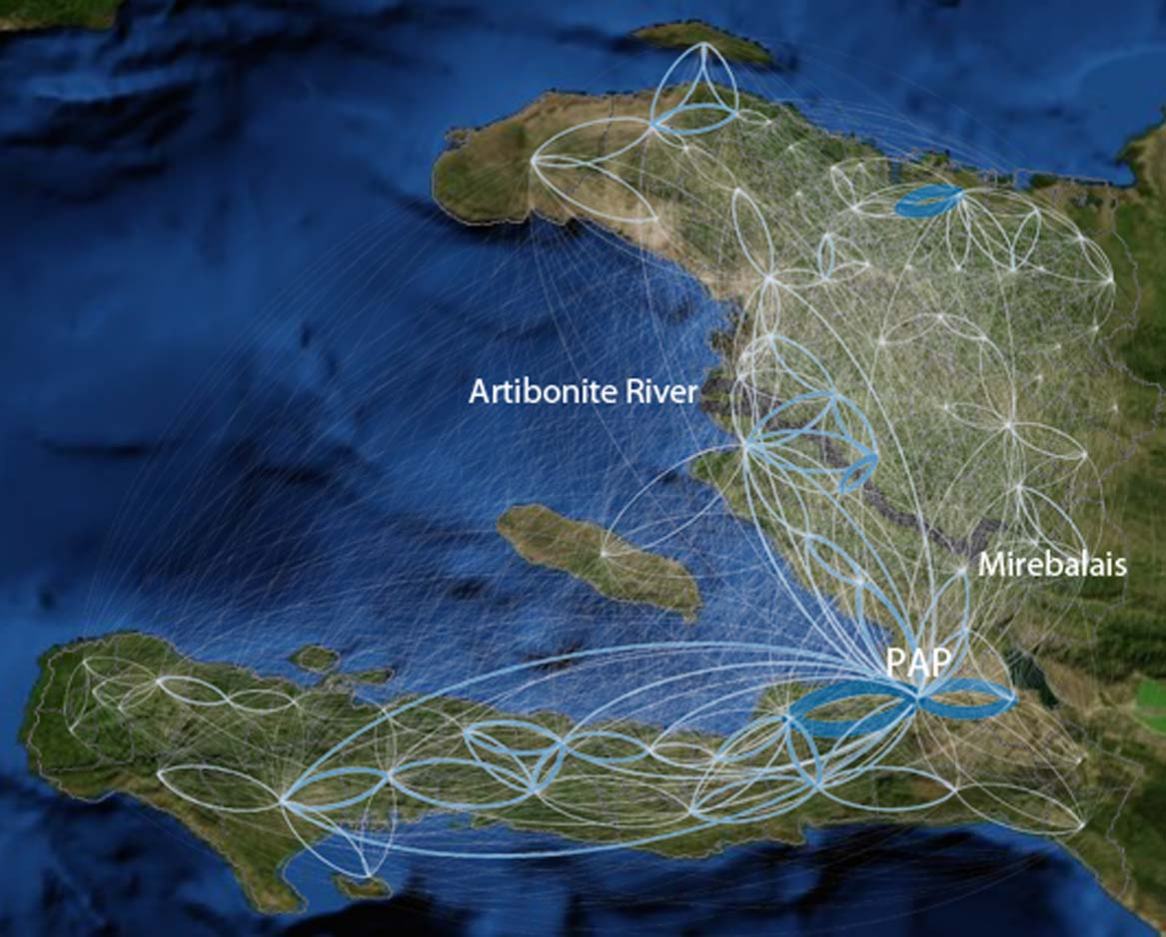
Mobile phone mobility network. The average absolute number of mobile phones moving between the study areas (October 15 to December 19, 2010). Thicker, bluer lines indicate larger number of travelers. The original outbreak location (Mirebelais), the Artibonite River (dark blue) and Port-au-Prince (PAP) are depicted (visualisation using Gephi and ArcGIS).

We used the mobile phone data to construct a mobility matrix *M^phone^*, with elements 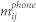, indicating the average daily proportion of mobile phones relocating from study area *i* to *j*, comparing their last registered location on day *t* with their last registered location on day *t-1*. The mobility network built on the basis of *M^phone^* displays strong connectivity both between Port-au-Prince and large parts of the country as well as between other urban areas and their surrounding countryside (Fig. 1). We calculated the infectious pressure *P_j_*(*t*), sustained by each study area *j* during the period from October 21 (from seven days after the disease onset of the first case in Haiti) to December 16, according to Eq. 1, in which *c_i_*(*t*) is the number of reported cases in study area *i* on day *t*:
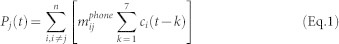


We thus assumed that a) the number of infectious individuals in a study area was proportional to the cumulative number of reported cases in the area during the preceding seven days (approximating the generation time of cholera, see also S5 and below)^29^,^30^ and b) the proportion of mobile phone movements between study areas was representative of the movements of infectious persons between study areas.

For comparative purposes, we implemented a gravity model of population mobility. Gravity models have previously been used to model mobility of infectious persons in a large number of studies in Haiti and elsewhere^8^,^16^,^17^,^31^ and assume that population mobility between areas depends positively on their population sizes and negatively on the distance between them. We calculated the infectious pressure according to Eq. 1, replacing 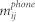 by 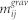 using the following gravity model^31^. 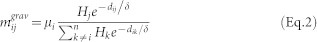
in which *μ_i_* is the average daily proportion of the population in area *i* that moves out of the area. The remaining ratio is the estimated probability that an individual leaving study area *i*, goes to study area *j*. *H_j_* denotes the population in study area *j*, and *d_ij_* denotes the distance between the population-weighted centroids of study areas *i* and *j*.

**Figure 2:**
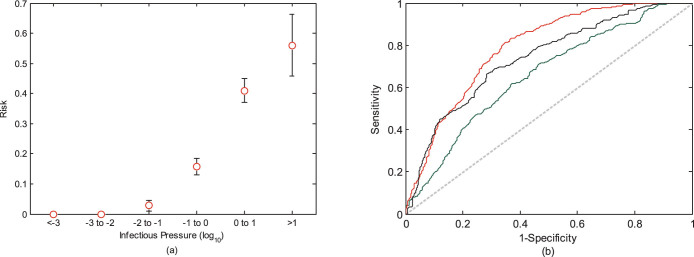
(a) Relationship between infectious pressure, calculated from the mobile phone data (*P^phone^*), and the risk of areas experiencing a new outbreak within seven days. Ninety-five percent confidence intervals based on a binomial distribution are included. (b) ROC curve (sensitivity and specificity) for predicting outbreak occurrence within seven days at increasing thresholds of infectious pressure (red: *P^phone^*, green: *P^grav^*^1^, black: *P^grav^*^2^). Random guesses would yield values along the diagonal line.

In the absence of detailed mobility data, values for *μ_i_* and *δ* (the scaling parameter) in Eq. 2 are unknown and needs to be assigned. Parameter values vary however widely between studies. As appropriate values for Haitian mobility are unknown, we chose to optimize the model based on the retrospective case data from the complete study period. Note that this optimisation thus could not have been performed until after the spatial spread of the epidemic was complete. Our comparison model thus performs better than a model that could have been developed during the epidemic. We produced two separate optimisations. In the first we optimised the gravity model by choosing values for *μ_i_* and *δ* (0.154 and 122 respectively), which minimised the residual sum of squares between reported daily cholera cases in each study area and the estimated pressure from the gravity model^31^. In the second we chose parameter values (0.158 and 3.5 respectively) that maximised the area under the curve (AUC), among all possible ROC curves. The ROC curve in this analysis depicts the sensitivity and specificity, using increasing thresholds of infectious pressures, to predict outbreak occurrence^32^ (see Results and Fig. 2b). We denote infectious pressures calculated from mobile phone movements by *P^phone^* and from the optimised gravity models by *P^grav^*^1^ and *P^grav^*^2^, respectively (see S3).

## Results

### Outbreak risk

For an efficient response to a developing epidemic, it is important to rapidly focus intervention resources to areas at highest risk. By utilizing data for all days of the study period, we plotted the proportion of non-infected communes that experienced an outbreak within seven days, for various intervals of infectious pressure, *P^phone^* (Fig. 2a). The risk of a study area experiencing a new outbreak correlated closely with the infectious pressure. Over a pressure level of 22 (*P^phone^*), all areas (six study areas) experienced outbreaks within seven days (see S5 for *P^grav^*^1^, *P^grav^*^2^ and sensitivity analyses).

### Sensitivity and specificity of outbreak predictions

Building upon this strong correlation between infectious pressure and outbreak risk, we created a binary test to predict an outbreak occurring within the upcoming seven days, based solely on thresholds of infectious pressure. We predicted an outbreak to occur at a given pressure threshold) and plotted the corresponding sensitivity and specificity of each threshold (Fig. 2b). We compared the model based on the mobile operator mobility data (*P^phone^*) with the gravity models (*P^grav^*^1^ and *P^grav^*^2^), for which *P^grav^*^2^ was optimised specifically to yield the maximum possible area under the curve (AUC) in this analysis. Comparing these ROC curves, the *P^phone^* model clearly performs better than the *P^grav^*^1^ model and slightly better than the *P^grav^*^2^ model, yielding a higher specificity for a given level of sensitivity. Note that both gravity models rely on parameter optimisations that could not have been performed until the epidemic spread was completed. Analyses of ranks of infectious pressure yielded similar results (S4). As the generation time of cholera in Haiti is uncertain and may have deviated from the seven-day period assumed in this study (Eq. 1)^29^, we additionally calculated infectious pressure based on other time periods (three, five and nine days), which did not alter the results (S5).

### Early outbreak incidence

In addition to predicting the risk of a new outbreak occurring in an area, an effective health care response requires good estimates of the number of cases that are likely to occur if an outbreak takes place. We may however expect the local evolution of an epidemic, after its start, to be largely dependent on local environmental and behavioural factors. One may thus hypothesize that the infectious pressure leading to the seeding of an outbreak would have little further influence on the number of cases in an area. This did however not seem to be the case.

**Figure 3:**
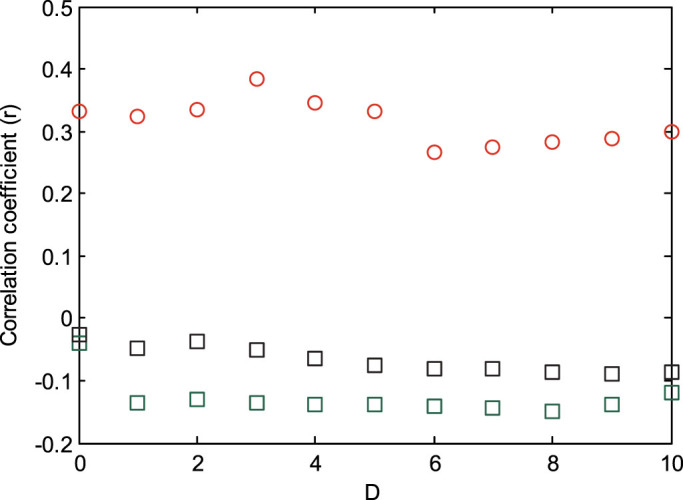
Correlation (r) between infectious pressure at outbreak onset and average daily number of reported cases during the first *D* days of the outbreak (one to ten days from onset). Red: *P^phone^*, solid green: *P^grav^*^1^, black: *P^grav^*^2^.

We correlated, for each newly infected area, the infectious pressure sustained by the area at outbreak onset with the average daily number of cases during the first *D* days of the new outbreak. For all values of *D* from one to ten days, we found a linear correlation (r) of approximately 0.3 (Fig. 3). Correlation coefficients were significant (p < 0.05) for all periods for the *P^phone^* model and non-significant for all 10 periods for the *P^grav^*^1^ and the *P^grav^*^2^ model (one outlier excluded).

## Discussion

Our results show that the risk of epidemic onset of cholera in a given area and the initial intensity of local outbreaks could have been anticipated during the early days of the Haitian epidemic using case reports and the mobility patterns of mobile phones. We show that the specificity and sensitivity of predictions of epidemic spread was improved or comparable to currently available optimized mobility models. Most importantly, the predictions based on the mobile operator data did not rely on retrospective optimization of parameter models and could thus be available from the start of an outbreak. This is important as gravity model parameters are highly context specific^33^,^34^. These results indicate that outbreak preparedness and response to epidemic agents, such as cholera, can be enhanced. The findings may have particular importance for improving early containment efforts of emerging infectious diseases, such as high mortality strains of pandemic influenza, and the response to vaccine-preventable diseases, such as measles, in low-income settings.

Cholera is known to be disseminated not only via human movement, but also by water and sometimes food contamination^35^. Even stronger predictive power may thus be achieved for infectious agents that exhibit exclusive person-to-person transmission. Furthermore, in this study, we focused on an extremely large and rapid cholera epidemic. It is likely that surveillance data would be more accurate for a disease that exhibits obvious symptoms and longer generation times, which may reduce reporting bias and delays. In particular, mobile operator data may represent a powerful tool for the containment of measles, which exhibits a high mortality rate, is preventable with vaccine, is readily identifiable based on simple clinical criteria and displays a sufficiently long generation time. Mobile phone-based connectivity matrices may also be very useful for early containment of emerging infectious agents, such as the localized appearance of high-mortality influenza strains with pandemic potential^6^. Mobile operator data may be especially advantageous in settings where poor road quality renders distance a weak measure of connectivity.

Access to operator data is a prerequisite for the future use of the method. The study demonstrates however that for the purpose of predicting future outbreaks, mobile phone operators may not need to provide access to their complete customer databases, but rather only to aggregated data on mobility between areas. Such aggregated connectivity matrices can be made available for preparedness purposes before outbreaks. They can then be repeatedly updated during the outbreak response to take into account changes in population mobility, which would not be captured by a gravity model.

In newly infected areas, infectious pressure based on the mobile phone model (but not gravity models) correlated positively with initial incidence. The correlation between mobility and initial incidence was unexpected as cholera transmission depends on a number of local socio-economic and environmental conditions. There may either be a causal connection between mobility and outbreak size (if rapid outbreaks were caused by multiple seeding events), alternatively areas between which there is high mobility may have environmental and behavioural similarities. Although the findings indicate an important new use of mobility data, a policy relevant tool for predicting early case numbers thus needs to incorporate additional variables to strengthen correlations.

Some study limitations should be noted. Reporting errors and delays are likely to have occurred in the case data and may have reduced the predictive accuracy for both the phone and gravity models. This will also be the case in future applications of the method. The case data in the study should be interpreted as reflecting relative rather than absolute differences in case load per area, as the Haitian cholera reporting system excluded asymptomatic and mild infections, as well as some severely ill patients who did not reach health facilities^36^.

Short-range movements may be under-recorded in the mobile data since they take place over shorter time intervals. However, weighting short and long-term movements differently did not change the prediction results (S5). Frequency of mobile phone use varies throughout the country and differential mobility between phone and non-phone users may exist. Studies evaluating bias in mobility estimates based on mobile operator data do however provide strong support for operator data being the currently best measure of nationwide mobility patterns^27^,^28^,^37^. Although this study focuses only on the influence of human mobility, future mobile phone based models focusing on cholera may benefit from including data on spatial distributions of access to water and sanitation^38^, bacterial transmission via waterways^31^, agricultural practices^39^, differential population immunity levels^40^ and interactions between infectiousness and mobility and between infectiousness and phone use.

In summary, the results show that mobile phone mobility patterns in Haiti during the 2010 cholera outbreak enabled predictions of epidemic spread, which did not require retrospective optimization of parameter models and could thus be available at outbreak onset. The results imply that anonymous mobile phone data may represent a key data source to both increase our understanding of the mechanisms underlying the spatial spread of infectious agents and provide an important policy-relevant tool for future outbreak preparedness and response efforts.

## Author Contributions

L.B. wrote the main manuscript text and all authors provided edits. X.L., L.B., R.P., K.S. and J.G. performed analyses. E.W., S.M. and S.R. provided additional input and participated in data collection. X.L. prepared the figures.

## Supplementary Material

Supplementary Information Supplementary Information
