# Supplementary material for: Using Mobile Phone Data to Predict the Spatial Spread of Cholera
Source: Sci Rep. 2015 Mar 9;5:8923. doi: 10.1038/srep08923 (PMC4352843; doi:10.1038/srep08923)
Supplement: Supplementary Information [file srep08923-s1.pdf]

# Supplementary Information:

## Using Mobile Phone Data to Predict the Spatial Spread of Cholera

***Linus Bengtsson[a,b], Jean Gaudart[c], Xin Lu[d,a,b], Sandra Moore[e], Erik Wetter[b,f], Kankoe Sallah[c], Stanislas Rebaudet[e], and Renaud Piarroux[e]***

[a]*Department of Public Health Sciences. Karolinska Institutet, Stockholm, Sweden;*
[b]*Flowminder Foundation, Stockholm, Sweden;*
[c]*Aix-Marseille University, UMR 912 SESSTIM (INSERM-IRD-AMU), Marseille, France;*
[d]*College of Information System and Management, National University of Defence Technology, Changsha, China;*
[e]*Aix-Marseille University, UMR MD 3, Marseille, France;*
[f]*Stockholm School of Economics, Stockholm, Sweden;*

# S1 Epidemic development and study areas

## A. Epidemic Curve

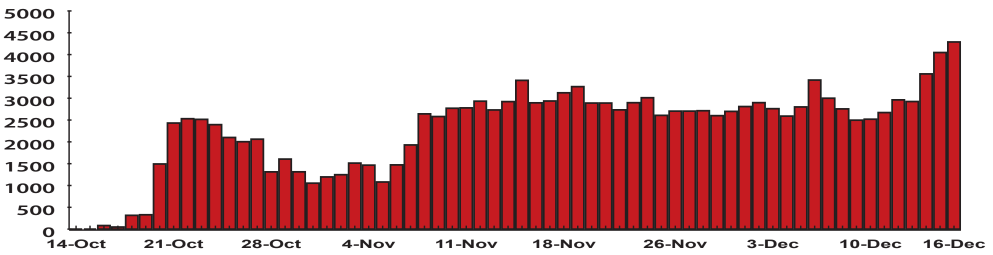


*Figure S1: Daily number of cases reported nationally from October 14 to December 16, 2010.*

## B. Study period and study area

The 2010 Haitian outbreak was the largest cholera epidemic to strike a single country in recent history.[1] As of November 13, 2013, the Haitian cholera epidemic had caused 8,448 deaths, and 689,448 cases had been registered.[2]

The outbreak commenced with contamination of the Artibonite River by a *Vibrio cholera* O1 strain near Mirebalais, 60 km north of the capital Port-au-Prince. The first confirmed cholera case resided in Meille, a hamlet two miles south of Mirebalais, and developed symptoms on October 14, 2010.[3] By October 19, suspected cases (775 cases) were reported exclusively upstream in Mirebalais. The 20th of October marked an explosion of cases throughout the communes of the lower Artibonite River valley, where bacterial transmission occurred via the Artibonite River. In less than two months, the epidemic spread from the areas adjacent to the river, throughout the entire country.

We defined the end of the study period as the 16 December, the peak of the epidemic, when all but one out of the 140 communes had reported at least one case. Reporting from this remaining commune (Fonds-Verrettes) is questionable, as it had no recorded cases for a further 151 days (24 May 2011). Given that the case definition was broad (acute watery diarrhea irrespective of cause) this seems unlikely considering the normal panorama of cases appearing in local clinics in Haiti. We thus set the final date for the study period to 16 December and excluded Fonds-Verrettes from the analyses.

Due to the massive number of patients, Haitian authorities decided to base the cholera surveillance system on institutional data (cases per health facility) as it was not realistic to track the high number of individual patient addresses from hospital register books. In communes without cholera treatment centres (CTCs) symptomatic individuals are known to have sought health care in neighbouring communes. Before analyses started, and to err on the safe side, we thus merged communes without a CTC with the closest commune in which a CTC was present. To further ensure that this approach reflected actual health seeking behaviour, we reviewed transportation times to CTCs and performed numerous field investigations of CTCs, including review of addresses in the patient records.[4]

The Port-au-Prince metropolitan area is a special case. Although it would have been valuable to follow the development of the epidemic in this area, we chose to aggregate these into one study area, as it was clear in review of patient records that cross-commune health seeking in this area had been extensive. Seventy-eight study areas were in this way created from the one hundred forty Haitian communes. Definitions of study areas were set before analyses started and were not subsequently changed. We cannot exclude the possibility that health seeking in some cases would have taken place across our study areas but this should have been rare and we believe that it is unlikely that such events would have seriously biased the results.

## S2 Mobile operator dataset

Haiti had at the time of the cholera outbreak two GSM mobile phone network operators. The analysed anonymised mobile operator data belongs to the largest mobile phone operator in Haiti (Digicel Haiti). We have not found evidence of differences in the characteristics of the two companies' subscriber bases. Digicel Haiti's market share at the time of the study was approximately 60%. Digicel Haiti's network covered at the time 90% of the inhabited areas.[5] The ratio between the number of active SIM cards in the country and the total population all ages was at the time 35% (grown to 61% in 2013).[6]

We assigned towers to the respective study areas. Tower density varies greatly with population density, with towers placed especially close in urban areas. The maximum distance for any pair of adjacent towers was 38.6 km and the median number of towers per study area was 4. The anonymous mobile phone data analysed included the last outgoing call and text message for each subscriber, for each day, from October 15 to December 19, 2010. We created a mobility network based on the individual anonymous trajectories by calculating for each pair of study areas, the proportion of mobile phones in area i on day t located in area j on day t+1.

## S3 Implementation of Gravity Models

Eq. 2 (main paper) gives the equation for the gravity model. Due to the geographic shape of Haiti (Fig. 1a main article), a straight line between two study areas would often cross bodies of water. We therefore calculated the distance between study areas $i$ and $j$ by the shortest paths of the Euclidean distance over a spatial network, which was constructed by placing nodes in the population-weighted centroid[7] of each study area and connecting each node with its four nearest neighbouring nodes.

Two parameters ($\mu_i$ and $\delta$) in Eq. 2 need to be assigned. There are multiple ways these can be optimized and we have chosen two approaches. First we chose values for $\mu_i$ and $\delta$ that, for each day during the study period, minimized the residual sum of squares between reported daily cholera cases in each study area and the estimated pressure from the gravity model.[8] This optimization yielded a $\mu_i$ of 0.154 and a $\delta$ of 122 ($P^{grav1}$).

Second we chose parameters to maximize the area under the curve (AUC) in the analysis of Fig. 2b. Given that $\mu_i$ is assumed to be the same for all areas, only values of $\delta$ will affect the AUC. Maximum AUC was found with a $\delta$ of 3.5 ($P^{grav2}$). Below we show how AUC changes over increasing values of $\delta$.

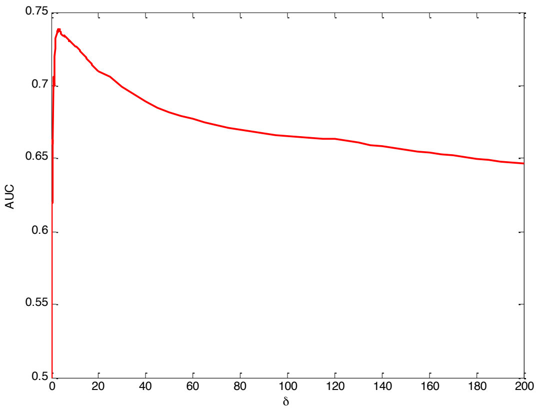


*Figure S2: AUC, as defined in the main paper, according to choice of gravity model parameters.*

# S4 Rank Analyses

We show below analyses complementary to those displayed in Figures 2 in the main article but based on ranks of infectious pressure instead of absolute values of pressure.

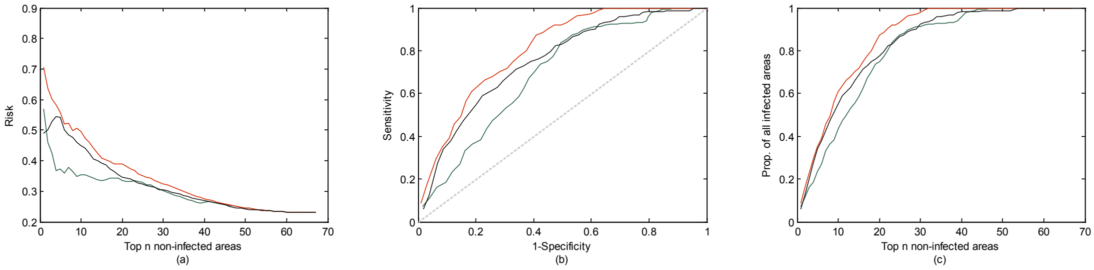


*Figure S3: a) Risk of a new outbreak within seven days for the top n non-infected communes with highest pressure. b) ROC curve based on ranks (i.e. the sensitivity and specificity of predicting an outbreak to occur within seven days for increasing thresholds of rank of infectious pressure). c) Proportion of all actually infected areas (seven days later) that were included among the top n areas with highest pressure. Red: $P^{phone}$; Green: $P^{grav1}$; Black: $P^{grav2}$.*

Below we show the accuracy of ranked analyses over time.

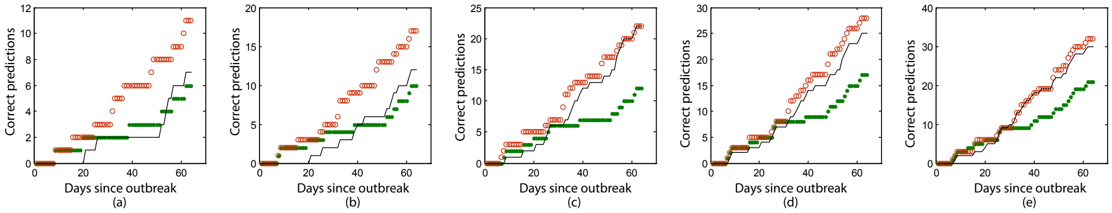


*Figures S4a-e: Study areas were, for each day, ranked according to their infectious pressures. The cumulative number of newly infected areas (seven days later) that were included among the top n areas with highest pressure are shown for n=1,2,3,4,5 (a-e). As n grows and predictions become less and less specific, the models converge. Red:* $P^{phone}$*; Green:* $P^{grav1}$*; Black:* $P^{grav2}$.

# S5: Sensitivity Analyses

## A. Definition of infectious pressure

Infectious pressure sustained by each study area was calculated based on the mobility pattern between areas and the cumulative number of cases during the preceding seven days, within contributing study areas, according to Eq. 1 (main article) as follows:

$$P_j(t) = \sum_{i,i\neq j}^{n} \left[ m_{ij}^{phone} \sum_{k=1}^{7} c_i(t-k) \right],$$

where n is the total number of studied areas, $m_{ij}^{phone}$ is the average daily proportion of mobile phones relocating from study area i to j $\left(\frac{\text{relocating phones}_{i\to j}}{\text{phone holders}_i}\right)$ and $c_i(t)$ is the number of reported cases in study area i on day t. We assumed that $\sum_{k=1}^{7} \frac{c_i(t-k)}{H_i}$ is proportional to the prevalence of infectious persons in area i on day t.

As the generation time of cholera is not well characterized for the Haitian epidemic we performed a sensitivity analysis calculating the infectious pressure based on periods of three, five, seven and nine historical days. The results remained stable (Fig. S5).

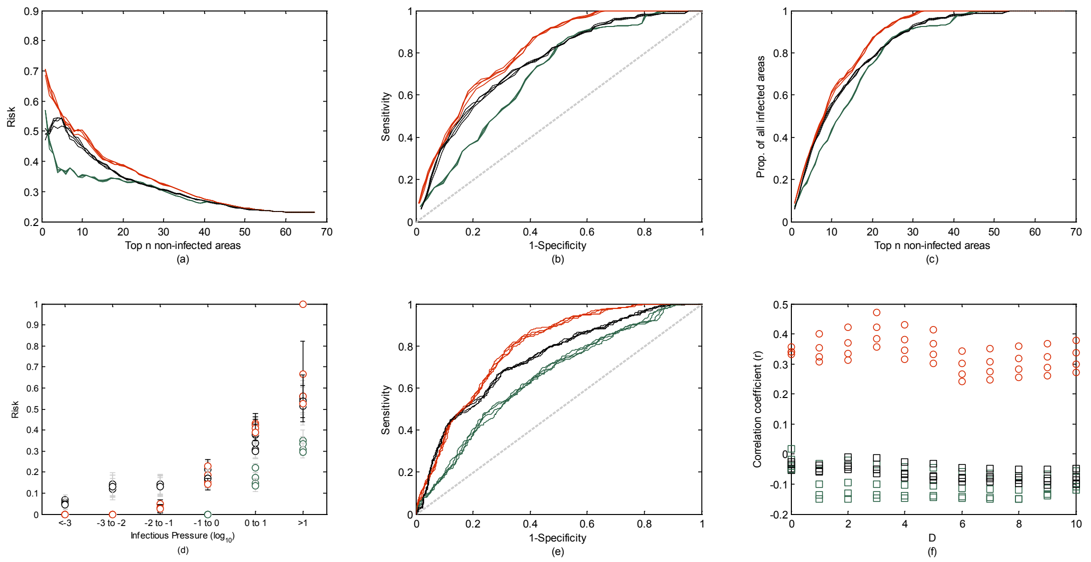


*Figures S5: a-c) Versions of fig. S2 with infectious pressure calculated based on historical periods of three, five, seven and nine days. d-f) Versions of fig. 2 and 3 (main article) with infectious pressure calculated based on historical periods of three, five, seven and nine days. Red:* $P^{phone}$; *Black:* $P^{grav1}$; *Green:* $P^{grav2}$.

## B. Outbreak Definition

From some study areas, isolated cases were reported early on in the epidemic, although ensuing cases were not reported for several weeks. According to the case definition ("acute watery diarrhea, with or without vomiting, in persons of all ages"), some reported suspected cases (with no biological confirmation) are thus likely to have been due to other agents. For analyses in the main paper we defined a new cholera outbreak to have taken place if a study area reported at least five cases on any given day. Figure S6 displays the results obtained when we gradually decease the minimum number of cases (seven, five, three and one case on any one day) required to define that an outbreak have occurred. When more than a single case is required to define an outbreak, the results are stable.

*a)*

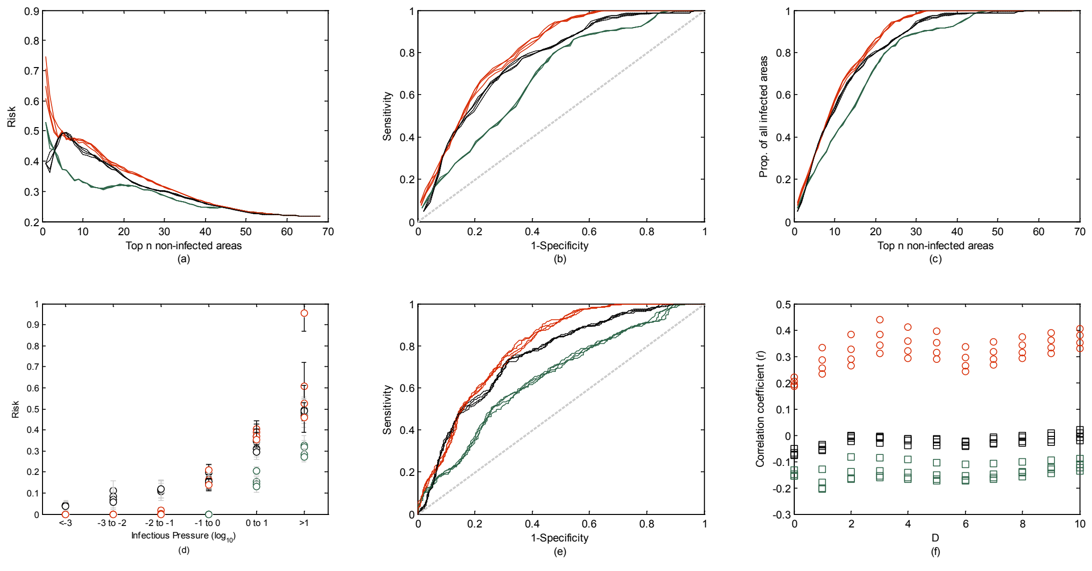

*b)*

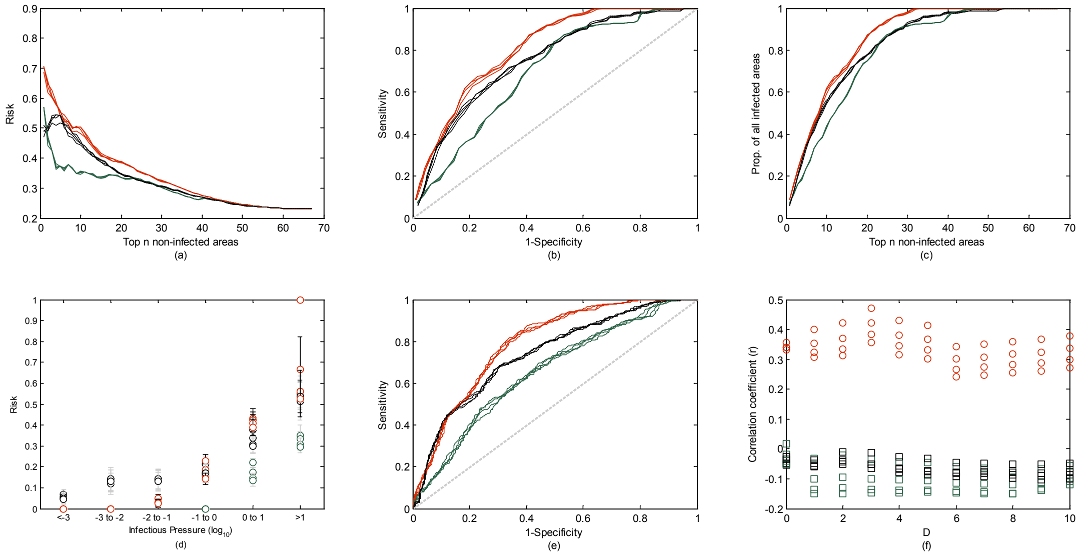
Risk
Top n non-infected areas
(a)
Sensitivity
1-Specificity
(b)
Prop. of all infected areas
Top n non-infected areas
(c)
Risk
Infectious Pressure (log10)
<-3
-3 to -2
-2 to -1
-1 to 0
0 to 1
>1
(d)
Sensitivity
1-Specificity
(e)
Correlation coefficient (r)
D
(f)

c)

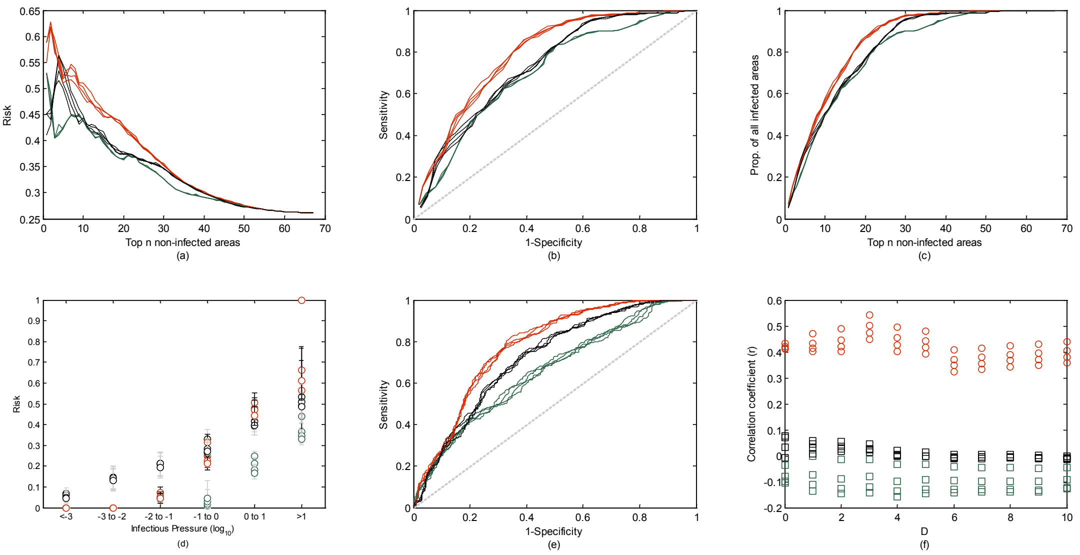
Risk
Top n non-infected areas
(a)
Sensitivity
1-Specificity
(b)
Prop. of all infected areas
Top n non-infected areas
(c)
Risk
Infectious Pressure (log10)
<-3
-3 to -2
-2 to -1
-1 to 0
0 to 1
>1
(d)
Sensitivity
1-Specificity
(e)
Correlation coefficient (r)
D
(f)

*d)*

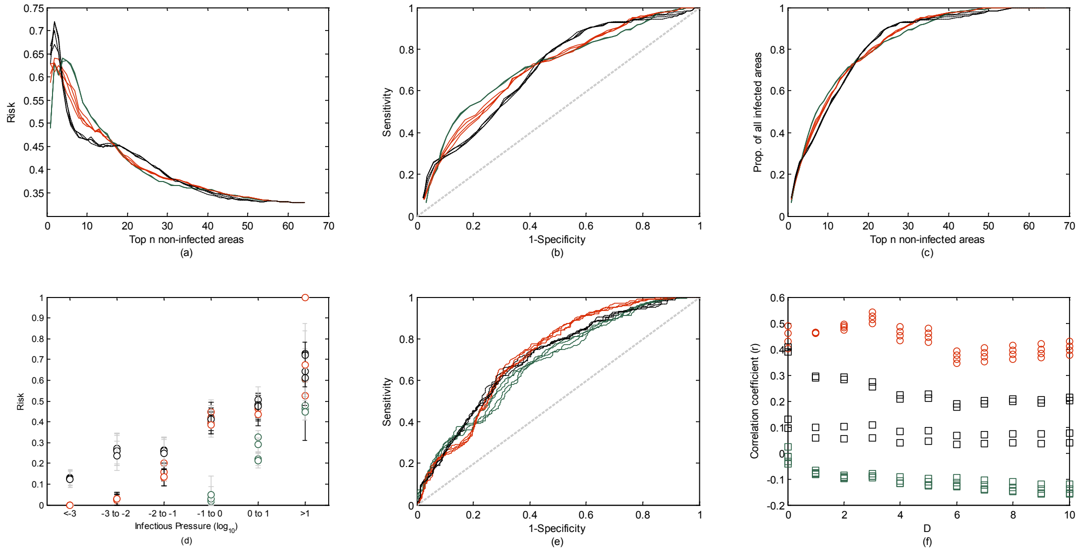
Risk
Top n non-infected areas
(a)
Sensitivity
1-Specificity
(b)
Prop. of all infected areas
Top n non-infected areas
(c)
Risk
Infectious Pressure (log10)
<-3
-3 to -2
-2 to -1
-1 to 0
0 to 1
>1
(d)
Sensitivity
1-Specificity
(e)
Correlation coefficient (r)
D
(f)

*Figures S6a-d: Four different versions of fig. S5 with the outbreak definitions based on a decreasing number of reported cases for any given day and study area. a) seven or more reported cases, b) five or more reported cases, c) three or more reported cases and d) one or more reported case.*

## C. Potential under sampling of short trips

For a given person, short trips are more likely to be under-recorded in the mobile data since they take place over shorter time intervals. We checked the robustness of the predictions by weighting up short and long trips respectively. To minimize bias furhter below we additionaly weighted flows between study areas by the proportion of each tower area that covered the respective study areas. Results are highly robust.

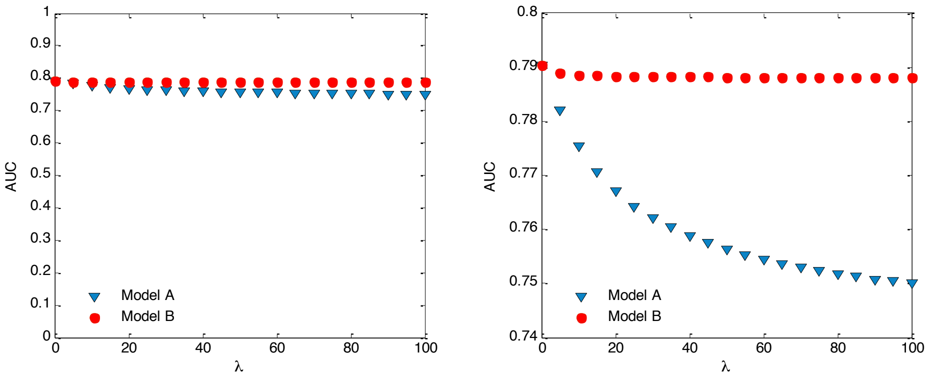


*Fig S7 a: Change of AUC with differential weighting of short and long trips. Model A weights up short distance movements. The shortest travel in model A counts λ times more than the longest travel, in the calculation of infectious pressures. The weight, $w_1 = \lambda$ *(maxdistance-distance(tower i, tower j))/(maxdistance-mindistance). Model B weighs up long distance movements. The longest travel counts λ times more than the shortest travel in the calulation of infectious pressure. The weight, $w_2 = \lambda$ *(1-(maxdistance-distance(tower i, tower j))/(maxdistance-mindistance)); b) Same figure as a) but zoomed in on the Y-axis.*

**References**

1. Barzilay, E. J. et al. Cholera surveillance during the Haiti epidemic--the first 2 years. The New England journal of medicine **368**, 599-609, doi:10.1056/NEJMoa1204927 (2013).
2. Rebublique d'Haiti. Ministère de la Santé Publique et de la Population. <http://mspp.gouv.ht/newsite> (2013).
3. Piarroux, R. et al. Understanding the cholera epidemic, Haiti. Emerg Infect Dis **17**, 1161-1168, doi:10.3201/eid1707.110059 (2011).
4. Gaudart, J. et al. Spatio-temporal dynamics of cholera during the first year of the epidemic in Haiti. PLoS Negl Trop Dis **7**, e2145, doi:10.1371/journal.pntd.0002145 (2013).
5. Bengtsson, L., Lu, X., Thorson, A., Garfield, R. & von Schreeb, J. Improved response to disasters and outbreaks by tracking population movements with mobile phone network data: a post-earthquake geospatial study in Haiti. PLoS Med **8**, e1001083, doi:10.1371/journal.pmed.1001083 (2011).
6. GSMA. Mobile for Development Intelligence - Haiti, <https://mobiledevelopmentintelligence.com/countries/HTI-haiti#> (2014).
7. U.S. Census Bureau. Demobase-Gridded Mapping, Haiti, <http://www.census.gov/population/international/data/mapping/demobase.html>
8. Rinaldo, A. et al. Reassessment of the 2010-2011 Haiti cholera outbreak and rainfall-driven multiseason projections. Proceedings of the National Academy of Sciences of the United States of America **109**, 6602-6607, doi:10.1073/pnas.1203333109 (2012).
